# Epidemiological analysis of intra-abdominal infections in Italy from the Italian register of complicated intra-abdominal infections—the IRIS study: a prospective observational nationwide study

**DOI:** 10.1186/s13017-025-00590-x

**Published:** 2025-03-17

**Authors:** Federico Coccolini, Etrusca Brogi, Marco Ceresoli, Fausto Catena, Angela Gurrado, Francesco Forfori, Lorenzo Ghiadoni, Ettore Melai, IRIS study group, Camila Cremonini, Serena Musetti, Luigi Cobuccio, Ismail Cengeli, Dario Tartaglia, Filippo Vagelli, Giuseppe Zocco, Silvia Strambi, Francesco Arces, Alice Salamone, Rossella Facchin, Riccardo Guelfi, Jacopo Giuliani, Rachele Monetti, Massimo Chiarugi, Alessandro Cipriano, Francesco Corradi, Angelo Baggiani, Caterina Rizzo, Carmelo Mazzeo, Eugenio Cucinotta, Angela Gurrado, Mario Testini, Vittoria Giovane, Francesco Prete, Alessandro Pasculli, Gianluca Costa, Alessio Mazzoni, Davina Perini, Alessandra Risso, Andrea Spota, Alan Biloslavo, Alessandra Sguera, Marco Anania, Risso Alessandra, Carlo Vallicelli, Carlo Mazzucchelli, Giulia Ciabatti, Claudia Zaghi, Daniele Delogu, Dario Iadicola, Dario Parini, Daunia Verdi, Diego Visconti, Davide Luppi, Fabio Cavallo, Edoardo Ballauri, Elia Giuseppe Lunghi, Emanuele Doria, Fausto Rosa, Federica Chimenti, Fioralba Pindozzi, Francesca Sbuelz, Francesca Cammelli, Mario Herda, Francesca D’Agostino, Giacomo Carganico, Franco Badile, Giovanni Gambino, Giovanni Pirozzolo, Giuseppe Brisinda, Alberto Vannelli, Leonardo Andrea Delogu, Lorenzo Gamberini, Maria Grazia Sibilla, Matteo Nardi, Mauro Podda, Maximilian Scheiterle, Michela Giulii Capponi, Michele Malerba, Marco Milone, Luisa Moretti, Nicola Cillara, Noemi Di Fuccia, Pierpaolo Di Lascio, Pietro Fransvea, Sonia Agrusti, Mauro Santarelli, Stefano Piero Bernardo Cioffi, Stefania Cimbanassi, Michele Altomare, Francesco Virdis, Stefano Scabini, Beatrice Torre, Valentina Murzi, Francesco Salvetti, Paola Fugazzola, Nita Gabriela Elisa, Giovanni Bellanova, Monica Zese, Davide Luppi, Luigi Romeo, Andrea Muratore, Elia Giuseppe Lunghi, Rocco Scalzone, Stefano Perrone, Savino Occhionorelli, Francesca Gubbiotti, Rosa Scaramuzzo, Roberta Gelmini, Vincenzo Pappalardo, Filippo Paratore, Elena Adelina Toma, Fabio Benedetti, Massimo Sartelli

**Affiliations:** 1 General, Emergency and Trauma Surgery Department, Pisa University Hospital, Pisa, Italy; 2 Department Anaesthesia and Intensive Care, Pisa University Hospital, Pisa, Italy; 3 Neuroscience Intensive Care Unit, ASST Grande Ospedale Metropolitano Niguarda, Piazza Ospedale Maggiore, 3, 20162 Milan, Italy; 4 General Surgery Department, Milano Bicocca University Hospital, Monza, Italy; 5 General, Emergency and Trauma Surgery Department, Bufalini Hospital, Cesena, Italy; 6 General Surgery, Bari University Hospital, Bari, Italy; 7 Emergency Medicine Department, Pisa University Hospital, Pisa, Italy; 8 General Surgery Department, Macerata Hospital, Macerata, Italy

**Keywords:** Intra-abdominal infections, Antibiotic therapy, Surgery, Epidemiology, Antimicrobial stewardship

## Abstract

**Background:**

Intra-abdominal infections (IAIs) are common and severe surgical emergencies associated with high morbidity and mortality. In recent years, there has been a worldwide increase in antimicrobial resistance associated with intra-abdominal infections, responsible for a significant increase in mortality rates. To improve the quality of treatment, it is crucial to understand the underlying local epidemiology, clinical implications, and proper management of antimicrobial resistance, for both community- and hospital-acquired infections. The IRIS study (Italian Register of Complicated Intra-abdominal InfectionS) aims to investigate the epidemiology and initial management of complicated IAIs (cIAIs) in Italy.

**Material and method:**

This is a prospective, observational, nationwide (Italy), multicentre study. approved by the coordinating centre ethic committee (Local Research Ethics Committee of Pisa (Prot n 56478//2019). All consecutively hospitalized patients (older than 16 years of age) with diagnosis of cIAIs undergoing surgery, interventional drainage or conservative treatment have been included.

**Results:**

4530 patients included from 23 different Italian hospitals. Community Acquired infection represented the 70.9% of all the cases. Among appendicitis, we found that 98.2% of the cases were community acquired (CA) and 1.8% Healthcare-associated (HA) infections. We observed that CA represented the 94.2% and HA 5.8% of Gastro Duodenal perforation cases. The majority of HA infections were represented by colonic perforation and diverticulitis (28.3%) followed by small bowel occlusion (19%) and intestinal ischemia (18%). 27.8% of patients presented in septic shock. Microbiological Samples were collected from 3208 (70.8%) patients. Among 3041 intrabdominal sample 48.8% resulted positive. The major pathogens involved in intra-abdominal infections were found to be *E.coli* (45.6%). During hospital stay, empiric antimicrobial therapy was administered in 78.4% of patients. Amoxicillin/clavulanate was the most common antibiotic used (in 30.1% appendicitis, 30% bowel occlusion, 30.5% of cholecystitis, 51% complicated abdominal wall hernia, 55% small bowel perforation) followed by piperacillin/tazobactam (13.3% colonic perforation and diverticulitis, 22.6% cholecystitis, 24.2% intestinal ischemia, 28.6% pancreatitis). Empiric antifungal therapy was administered in 2.6% of patients with no sign of sepsis, 3.1% of patients with clinical sign of sepsis and 4.1% of patients with septic shock. Azoles was administered in 49.2% of patients that received empiric antifungal therapy. The overall mortality rate was 5.13% (235/4350). 16.5% of patients required ICU (748/4350). In accordance with mortality, it is important to highlight that 35.7% of small bowel perforation, 27.6% of colonic perforation and diverticulitis, 25.6% of intestinal ischemia and 24.6% of gastroduodenal complications required ICU.

**Conclusion:**

Antibiotic stewardship programs and correct antimicrobial and antimycotic prescription campaigns are necessary to ulteriorly improve the adequacy of drug usage and reduce the resistances burden. This will help in improving the care and the cure of the next generations.

## Background

Intra-abdominal infections (IAIs) are common surgical emergencies and have been reported as major contributors to non-trauma deaths in emergency surgical units worldwide [1]. Complicated IAIs (cIAIs) are those ones passing the visceral peritoneal barrier (e.g., abscesses, perforations). The cornerstone of effective treatment of cIAIs includes early recognition, adequate source control, appropriate antimicrobial therapy, and prompt physiologic stabilization using intravenous fluid therapy in critically ill patients [3–6]. Results from published clinical trials often may not be representative of the true morbidity and mortality rates of such severe infections. Guidelines helps in managing IAI but tailored management may differ according to local epidemiology [7].

The knowledge of the various bacteria epidemiology in the different regions is often impaired by the possibility to accrue data. Several data have been published about the topic enrolling patients from all around the world [8–11]. Dedicated studies as the present one may represent the starting point to diffuse the awareness about the necessity to improve knowledge about this important topic. National registries are needed, and they should be included in broader program such as the Web-based International Register of Emergency Surgery and Trauma (Wires-T). This will allow to accrue precise data with the possibility to sum or compare them.

In recent years, there has been a worldwide increase in infections caused by microorganisms resistant to multiple antimicrobial agents. This increase in antimicrobial resistance has been noted in both hospital and community settings. The increasing prevalence of multi-drug resistance is responsible for a significant increase in morbidity and mortality rates associated with intra-abdominal infections as well as a subsequent increase in overall healthcare costs [12–14]. Furthermore, a dramatic reduction in the development of new antibiotics effective against multidrug-resistant pathogens has further exacerbated the dilemma. Antibiotic stewardship program must be implemented at local and national base to optimize antibiotic usage and potentially reduce the overuse or misuse of antibiotics.

In fact, an antimicrobial-based approach in managing intra-abdominal infections always involves a delicate balance between the optimization of empirical therapy, which has been shown to improve clinical outcomes, and the reduction of excessive antimicrobial use, which has been proven to increase the rate of emergence of antimicrobial-resistant strains. To ulteriorly improve the quality of treatment a multidisciplinary approach (involving surgeon, infectious specialist, and intensive care specialist) is essential for the best management of such critical condition. It is crucial, moreover, that every clinician understand the underlying local epidemiology, clinical implications, and proper management of antimicrobial resistance, for both community- and hospital-acquired infections.

To the best of our knowledge, no previous studies have been designed and carried out with a purposely intention to investigate the Italian epidemiology of cIAIs. Furthermore, although previous studies analyzed prognostic factors in cIAIs [15–20], additional efforts should be made to identify further risk factors predictive of mortality in patients with cIAIs. Moreover, only few observational studies were published with the aim to investigate patient characteristics associated with a high risk of isolation of resistant pathogens from an intra-abdominal source [21–24]. The Italian Register of complicated Intra-abdominal InfectionS – the IRIS study aims to investigate the epidemiology and initial management of cIAIs in Italy.

## Methods

The IRIS study (Italian Register of Complicated Intra-abdominal InfectionS) is a prospective, observational, nationwide (Italy), multicentre study. The Study has been approved by the coordinating centre ethic committee (Local Research Ethics Committee of Pisa (Prot n 56,478//2019).

### Inclusion criteria

All consecutively hospitalized patients (older than 16 years of age) with diagnosis of cIAIs (defined as abdominal infections originating in an organ cavity, extending into the peritoneal space, and forming an abscess or peritonitis) undergoing surgery, interventional drainage or conservative treatment have been included in the study between May 1st, 2021 and April 31st, 2023.

### Data collection

Data were accrued prospectively in on-line case report platform (www.clinicalregisters.org).

The following data have been collected for each patient:

Demographic data (Gender, age). Antimicrobial therapy administered within one month prior to surgery, comorbidities (primary or secondary immunodeficiency, solid or haematopoietic and lymphoid malignancy, severe cardiovascular disease, chronic dialysis, history of MDRO colonization/infection). Clinical findings upon admission, as fever (defined as core temperature > 38.0° C) or hypothermia (core temperature < 36.0° C), leucocytosis (white blood count [WBC] > 12,000 cells/ml) or leukopenia (WBC < 4000 cells/ml), presence of localized pain, diffuse pain, abdominal rigidity [25, 26]. Patient clinical condition at admission. Setting of infection acquisition: complicated IAIs will be classified as community- acquired (CA-cIAIs) or healthcare-acquired (HA-cIAIs). Complicated IAIs will be considered as HA-cIAIs in patients hospitalized for at least 48 h during the previous 90 days; or those residing in skilled nursing or long-term care facility during the previous 30 days; or those who have received intravenous therapy, wound care, or renal replacement therapy within the preceding 30 days. Radiological diagnosis (ultrasound, radiological and computer tomography findings). Source of infection (stomach or duodenum, gallbladder, small bowel, colon, appendix or other), and peritonitis diffusion (generalized or localized peritonitis/abscess). Source control (conservative treatment, operative or non-operative interventional procedures) and its adequacy, defining the latter one as the achievement to establish the cause of cIAIs and to control the origin of peritonitis. Pre-operative antimicrobial prophylaxis or therapy (type of antimicrobial(s) administered, dosage, duration). Antimicrobial therapy performed (type of antimicrobial(s) administered, dosage, duration), specifying if empirical therapy or guided by antibiogram(s) performed. Clavien-Dindo Score [27]. Infectious post-operative complications (tertiary peritonitis, surgical site infections, pneumonia, bacteraemia, sepsis). Length of ICU stay. Length of hospital stay (LOS). In-hospital mortality. Cultures will be performed on intra-operative samples of peritoneal fluid or purulent exudate/discrete abscesses. The decision to perform cultures is according to the discretion of the providers for each patient with cIAIs. Microbiological data: Isolated microorganisms will be classified according to the joint recommendations for epidemiologic studies from the European Centre for Disease Prevention and Control, and from the Centers for Disease Control and Prevention.

### Microbiological data

Microbiological results to identify Gram-negative, Gram-positive and anaerobes bacteria, and fungi will be collected. Every hospital center determined antimicrobial susceptibilities of the isolated microorganisms according to its own procedures and criteria. Breakpoint guidelines used in antimicrobial susceptibility testing by each microbiology laboratory. All the microbiology laboratory used EUCAST guidelines [28, 29]. Isolated microorganisms will be classified according to the joint recommendations for epidemiologic studies from the European Centre for Disease Prevention and Control, and from the Centers for Disease Control and Prevention [30]. In our study, MDROs will be classified as follows: *E.coli* producing an extended-spectrum b-lactamase (ESBLp), *E.coli* resistant resistant to carbapenems, K. pneumoniae ESBLp, K. pneumoniae resistant to carbapenems, K. oxytoca ESBLp, K. oxytoca resistant to carbapenems, A. baumanii resistant to carbapenems, P. aeruginosa resistant to carbapenems, Methicillin-resistant Staphylococcus aureus (MRSA), E. faecalis resistant to vancomycin, E. faecium resistant to vancomycin, Bacteroides spp. resistant to metronidazole, Clostridium spp. resistant to metronidazole and C. albicans resistant to fluconazole.

### Statistical analysis

All analyses were performed using R Statistical Software (v4.1.2; R Core Team 2021). Bar graph and were obtained via the ggplot2 R package (v3.4.4; Wickham 2016). Italy Maps was obtained via the ggplot2 R package (v3.4.4; Wickham 2016), the tidyverse R package (v2.0.0; Wickham et al. 2019), rnaturalearth R package (v3.4; Massicotte and South 2023).

## Results

### Centre contribution

**Fig. 1:**
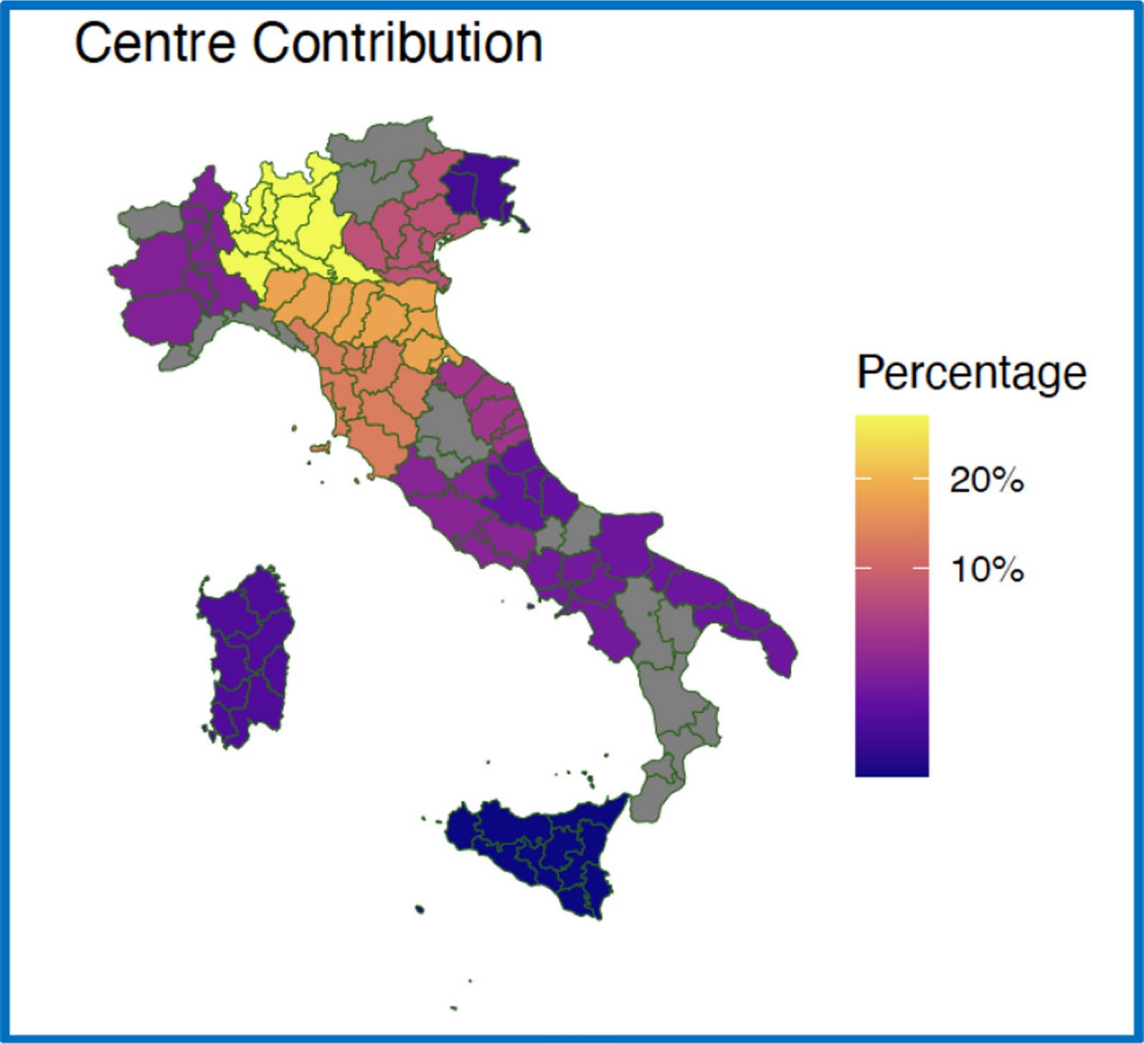
Geographical distribution of centre contribution to IRIS study. Italy map, geographical distribution of center contribution to database, percentage of cases for each region. All analyses were performed using R Statistical Software (v4.1.2; R Core Team 2021). Italy Maps was obtained via the ggplot2 R package (v3.4.4; Wickham 2016), the tidyverse R package (v2.0.0; Wickham et al. 2019), rnaturalearth R package (v3.4; Massicotte and South 2023)

We recruited a total of 4530 patients from 23 different Italian center. Centre contribution to database is shown in Fig. 1 according to regions; the region with the highest number of patients enrolled was Lombardy, which reported almost 1444 cases (31.8%) representing the most important contributor centre to database. Emilia Romagna recruited 894 cases (19.7%), followed by Toscana (641, 14.1%) and Veneto (389, 8.6%). No cases were reported from Liguria, Val d’Aosta, Trentino Alto Adige, Calabria, Molise, Abruzzo.

### Demographic characteristics

**Fig. 2:**
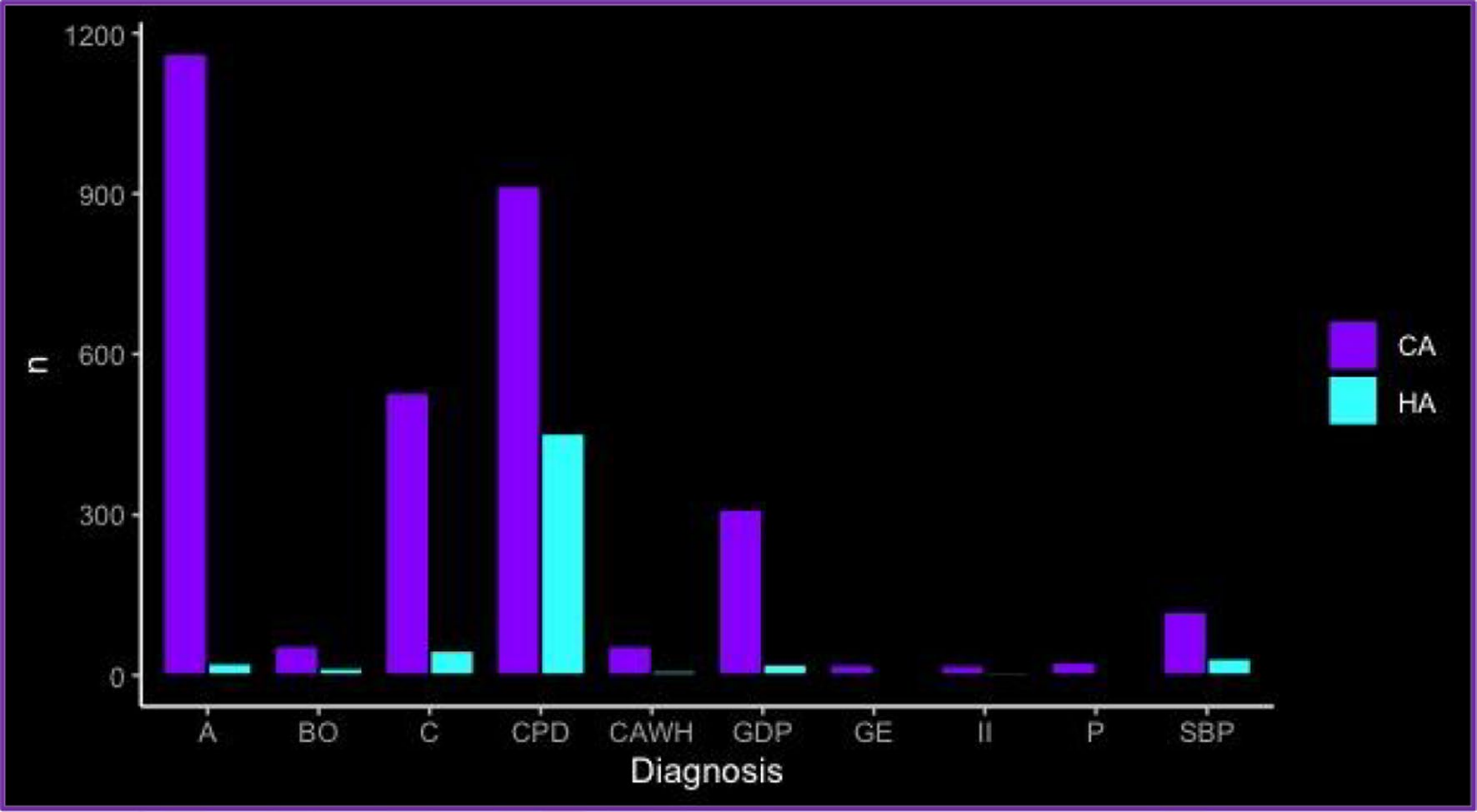
Number of cases of community acquired (CA) and healthcare associated (HA) intra-abdominal infection (IAI) in accordance with type of diagnosis. Bar graph: number of cases of community acquired and healthcare associated infection in accordance with type of diagnosis. All analyses were performed using R Statistical Software (v4.1.2; R Core Team 2021). Bar graphs were obtained via the ggplot2 R package (v3.4.4; Wickham 2016). A: Appendicitis; BO: Bowel Occlusion; C: Cholecystitis; CPD; Colonic perforation and diverticulitis; CAWH: Complicated Abdominal Wall Hernia; GDP: Gastro Duodenal Perforations; GE: Gynaecological Emergencies; II: Intestinal Ischemia; P: Pancreatitis; SBP: Small bowel perforation

Demographic characteristics of participants are shown in Table 1 according to Diagnosis. Community Acquired infection represented the 70.9% of all the cases and the predominant type of infection for all the diagnosis; 98.2% of appendicitis, 93.1% of cholecystitis, 71.7% of colonic perforation and diverticulitis, 94.2% of Gastroduodenal perforations and 91.6% of small bowel occlusion were community acquired Infections. Bar graph showing the number of cases of Community acquired and Healthcare Associated infection in accordance with type of diagnosis are presented in Fig. 2.

**Table 1:** Demographic characteristics and physiological status at presentation according to the type of diagnosis. (Data are presented as actual number (n), mean ± Standard deviation or percentage (%) where appropriate. F: Female; M: Male; ASA; American Society of Anaesthesiologists: Type of Infection, Community acquired “CA” vs Healthcare-associated “HA”: NA: not applicable.)

Diagnosis	n	Age	Sex	Type of infection	Septic status at presentation	n	%
			n	n (%)			
Appendicitis	1296 (28.6%)	39.2 ± 19.9	F	CA 1273 (98.2%)	No sign of sepsis	878	67.7
			555	HA 23 (1.8%)			
			M		Sepsis	400	30.9
			741		Septic shock	18	1.39
Cholecystitis	683 (15.1%)	64.8 ± 17.4	F	CA 744 (93.1%)	No sign of sepsis	446	65.3
			312	HA 47 (6.9%)			
			M		Sepsis	202	29.6
			371		Septic shock	35	5.1
Gastro duodenal perforations	361 (7.97%)	60.9 ± 18.6	F	CA 340 (94.2%)	No sign of sepsis	113	31.3
			135	HA 21 (5.8%)			
			M 226		Sepsis	202	55.9
					Septic shock	46	12.7
Bowel occlusion	166 (3.67%)	70.5 ± 15.7	F	CA 152 (91.6%)	No sign of sepsis	159	95.7
			77	HA 14 (8.4%)			
			M		Sepsis	5	3
			89		Septic shock	2	1.2
Small bowel perforation	168 (3.71%)	60.4 ± 20.5	F	CA 136 (81%)	No sign of sepsis	78	46.4
			91	HA 32 (19%)			
			M		Sepsis	52	30.9
			77		Septic shock	38	22.6
Colonic perforation and diverticulitis	1601 (35.3%)	64.5 ± 15.9	F	CA 1148 (71.7%)	No sign of sepsis	704	43.9
			773	HA 453 (28.3%)			
			M 828		Sepsis	638	39.8
					Septic shock	259	16.2
Intestinal Ischemia	39 (0.86%)	69.7 ± 18.5	F	CA 34 (82%)	No sign of sepsis	25	64.1
			17	HA 7 (18%)			
			M		Sepsis	5	12.8
			22		Septic shock	9	23.1
Gynecological emergencies	22 (0.48%)	37.3 ± 14.4	F	CA 20 (91%)	No sign of sepsis	8	36.4
			22	HA 2 (9%)	Sepsis	8	36.4
					Septic shock	6	27.2
Pancreatitis	57 (1.26%)	64.1 ± 16.7	F	CA 55 (98.3%)	No sign of sepsis	49	85.9
			80	HA 1 (1.7%)			
			M		Sepsis	3	8.8
			27		Septic shock	5	5.3
Complicated abdominal wall hernia	135 (2.98%)	70.1 ± 15.4	F	CA 126 (93.3%)	No sign of sepsis	78	46.4
			57	HA 9 (6.7%)			
			M		Sepsis	52	30.9
			78		Septic shock	38	22.6

The septic status at presentation were sepsis in 33.5% and septic shock in 27.8% of patients. In details, patients with clinical signs of septic shock were 16.2% of Colonic perforation and diverticulitis, 12.7% of gastroduodenal complications, 22.6% of small bowel perforation and 23.1% of Intestinal Ischemia (Table 1).

### Microbiology

Microbiological Samples were collected from 3208 (70.8%) patients (Table 2). 3041 (67%) were intrabdominal sample; 48.8% of intraabdominal samples resulted positive. In 50.4% of samples, we found that the intra-abdominal culture was associated with a positive blood stream sample (Table 2). The major pathogens involved in intra-abdominal infections were found to be *E.coli* (45.6%, as shown in Table 3). Only in 2.8% of the cases were collected both blood stream and intra-abdominal samples. In 0.9% of the cases only blood stream colures. Remarkably, in 29% of the cases microbiological samples were not collected. 3 Candida Glabrata and 142 Candida Albicans isolates were identified in Intraabdominal cultures. Even more, 29 non- albicans Candida and 6 non albicans candida resistant to fluconazole were found in the intra-abdominal cultures (Table 4).

**Table 2:** Microbiological sample and positive cultures

Microbiological sample	n	Positive cultures	N	%
Bloodstream & intra-abdominal cultures	127	Yes	64 (53 + 11 fungi)	50.4
		No	3	49.6
Intrabdominal cultures	3041	Yes	1484 (1301 + 183 fungi)	48.8
		No	1557	51.2
Bloodstream cultures	40	Yes	7	17.5
		No	33	2.5
Not performed	1321			

Data are presented as actual number (n), mean ± Standard deviation or percentage () where appropriate

**Table 3:** Microbiological samples & Gram – and Gram +

Microbiological sample	N of positive	Gram – and Gram +	n	%
Bloodstream cultures	7	Escherichia coli	4	57.2
		Multi	1	14.3
		Enterococcus	2	28.5
Intrabdominal cultures	1301	Acinetobacter baumannii	10	0.8
		Bacteroides	33	2.5
		Clostridium	7	0.5
		Escherichia coli	594	45.6
		Escherichia coli ESBL	44	3.4
		Enterobacter	49	3.8
		Klebsiella pneumoniae	56	4.3
		MDR	125	9.6
		Multi	209	16.1
		Proteus	16	1.2
		Pseudomonas aeruginosa	33	2.5
		Citrobacter freundii	1	0.07
		Serratia marcenscens	1	0.07
		Staphylococcus areus	34	2.6
		Staphylococcus capiti	1	0.07
		Staphylococcus epidermidis	4	0.3
		Staphylococcus hominis OXA-R	1	0.07
		Streptococcus	128	9.8
		Streptococcus constellatus	1	0.07
		Staphylococcus spp	1	0.07
Bloodstream & intra-abdominal cultures	53	Clostridium	2	3.8
		Escherichia coli	12	22.6
		Enterobacter	4	7.5
		Klebsiella pneumoniae	1	1.9
		MDR	5	9.4
		Multi	10	19
		Pseudomonas aeruginosa	3	5.7
		Enterococcus	9	16.9
		Staphylococcus areus	1	1.9
		Staphylococcus capitis	1	1.9
		Staphylococcus epidermidis	1	1.9
		Streptococcus	3	5.7
		Staphylococcus capitis	1	1.9

Data are presented as actual number (n), mean ± Standard deviation or percentage () where appropriate. MDR; Multidrug resistance: ESBL: Extended Spectrum Beta-Lactamase: Multi: association of antibiotic

**Table 4:** Microbiological samples & fungi

Microbiological samples	N of positive	Fungi	n	%
Intrabdominal cultures	183	Candida glabrata	3	1.6
		Candida albicans	142	77.6
		Candida albicans resistant to fluconazole	3	1.6
		Non-albicans candida	29	15.8
		Non-albicans candida resistant to fluconazole	6	3.2
Bloodstream & intra-abdominal cultures	11	Candida albicans	9	81.8
		Candida albicans resistant to fluconazole	1	9.1
		Non-albicans candida	1	9.1

Data are presented as actual number (n), mean ± Standard deviation or percentage () where appropriate

### Antibiotic usage

Most of the patient (93.2%) did not take antimicrobial therapy before admission to the hospital (Table 5). During hospital stay, empiric antimicrobial therapy was administered in 78.4% of patients. The kind of empiric antibiotic administered were listed in Table 6. Amoxicillin/clavulanate was the most common antibiotic used (in 30.1% appendicitis, 30% bowel occlusion, 30.5% of cholecystitis, 51% complicated abdominal wall hernia, 55% small bowel perforation) followed by piperacillin/tazobactam (13.3% colonic perforation and diverticulitis, 22.6% cholecystitis, 24.2% intestinal ischemia, 28.6% pancreatitis). Empiric antifungal therapy was administered in 2.6% of patients with no sign of sepsis, 3.1% of patients with clinical sign of sepsis and 4.1% of patients with septic shock (Table 6). Azoles was administered in 49.2% of patients that received empiric antifungal therapy (Table 7).

**Table 5:** Diagnosis & antibiotic therapy

Diagnosis	Total	Antimicrobial therapy in previous days	Duration of empiric antimicrobial therapy (days)	Empiric antibiotic	%
Appendicitis	1296	Yes 60 (4.6%)	6.4 ± 4.4	Amicasil	0.25
				Amoxicillin/clavulanate	30.6
				Cefepime	1
				Ceftazidime	0.5
				Ceftriaxone	3.5
				Ciprofloxacin	0.75
				Mefoxin	0.5
				Meropenem	1
				Moxifloxacin	1.3
				Multiple Antibiotics	18.1
				Piperacillin/tazobactam	7.1
				Azitromicine	0.25
				Cefazolina	0.25
				Cefoxitina	1.8
				Clindamicina	0.25
				Tigeciclina	0.5
		No 1236 (95.4%)			
Bowel occlusion	166	Yes 12 (7.3%)	7.2 ± 3.0	Amoxicillin/clavulanate	30.6
				Ceftazidime	1.02
				Ceftriaxone	1.02
				Ciprofloxacin	1.02
				Meropenem	1.02
				Multiple Antibiotics	40.8
				Piperacillin/tazobactam	23.5
				Tigeciclina	1.02
		No 154 (92.7%)			
Cholecystitis	683	Yes 68 (9.9%)	7.4 ± 8.5	Amoxicillin/clavulanate	30.8
				Ampicillin Sulbactam	0.7
				Cefepime	1.05
				Ceftazidime	0.7
				Ceftriaxone	21.9
				Ciprofloxacin	2.4
				Levofloxacin	0.35
				Meropenem	0.7
				Metronidazole	0.35
				Multiple Antibiotics	18.05
				Piperacillin/tazobactam	22.6
				Cefoxitina	0.35
		No 615 (90.1%)			
Colonic perforation and diverticulitis	1601	Yes 127 (7.9%)	12.9 ± 22	Amoxicillin/clavulanate	6.35
				Ampicillin Sulbactam	0.35
				Ceftriaxone	0.7
				Ciprofloxacin	0.5
				Levofloxacin	0.5
				Meropenem	1.4
				Metronidazole	0.2
				Multiple Antibiotics	73.7
				Piperacillin/tazobactam	13.3
				Vancomicina	0.35
				Zerbaxa	0.2
				Cefazolina	0.35
				Tigeciclina	2.2
		No 1474 (92.1%)			
Complicated abdominal wall hernia	135	Yes 2 (1.5%)	5.9 ± 3.4	Amoxicillin/clavulanate	51
				Cefepime	6.5
				Ceftriaxone	3.3
				Ciprofloxacin	1.6
				Levofloxacin	1.6
				Multiple Antibiotics	29.5
				Piperacillin/tazobactam	4.9
				Cefazolina	1.6
		No 133 (98.5%)			
Gastro duodenal perforations	361	Yes 20 (94.5%)	9.1 ± 6.5	Amoxicillin/clavulanate	5.8
				Caspofungina	1.2
				Ceftriaxone	0.6
				Multiple Antibiotics	70
				Piperacillin/tazobactam	19.6
				Vancomicina	0.6
				Cefoxitina	0.6
				Tigeciclina	1.7
		No 341 (5.5%)			
Gynaecological emergencies	22	No 22 (100%)	11.4 ± 6.8	Multiple Antibiotics	100
Intestinal ischemia	39	Yes 9 (77%)	10.5 ± 5.4	Amoxicillin/clavulanate	15
				Ampicillin Sulbactam	3
				Ceftazidime	3
				Meropenem	3
				Multiple Antibiotics	39.3
				Piperacillin/tazobactam	24.2
				Tigeciclina	12
		No 30 (23%)			
Pancreatitis	57	Yes 5 (91.2%)	8.8 ± 7.3	Amoxicillin/clavulanate	23.8
				Ceftriaxone	9.5
				Meropenem	4.7
				Multiple Antibiotics	33.3
				Piperacillin/tazobactam	28.6
		No 52 (8.8%)			
Small bowel perforation	168	Yes 3 (1.8%)	11.7 ± 8.9	Amoxicillin/clavulanate	60
				Ceftriaxone	5
				Multiple Antibiotics	20
				Piperacillin/tazobactam	5
				Tigeciclina	10
		No 165 (98.2%)			

Data are presented as actual number (n), mean ± Standard deviation or percentage () where appropriate

**Table 6:** Septic status empiric antifungal therapy

Septic status at presentation	Total n	Empiric antifungal therapy administration	n	%
No sign of sepsis	2591	No	2532	97.4
		Yes	68	2.6
Sepsis	1519	No	1472	96.8
		Yes	48	3.1
Septic shock	418	No	401	95.8
		Yes	17	4.1

Data are presented as actual number (n), mean ± Standard deviation or percentage () where appropriate

**Table 7:** Type of empiric antifungal therapy

Empiric antifungal therapy administration	Total n	Empiric antifungal therapy	n	%
Yes	134	Azoles	66	49.2
		Other antimycotic	68	50.8

Data are presented as actual number (n), mean ± Standard deviation or percentage () where appropriate

### Outcome

The overall mortality rate was 5.13% (235/4350). Remarkably, 16.7% of small bowel perforation, 10.2% of intestinal ischemia, 9.4% of gastroduodenal perforations and 9.2% of colonic perforation and diverticulitis died. 16.5% of patients required ICU (748/4350). In accordance with mortality, it is important to highlight that 35.7% of small bowel perforation, 27.6% of colonic perforation and diverticulitis, 25.6% of intestinal ischemia and 24.6% of gastroduodenal complications required ICU.

## Discussion

IAIs are a diffuse cause of surgical emergencies all around the world [31]. They may encompass different grading of severity. From a mild self-limiting infection to a severe peritonitis associated to septic shock. Management of IAI must be multidisciplinary and several specialists should be involved.

In fact, despite the still high mortality, thanks to the multidisciplinary management, short-term survival from sepsis of abdominal origin has improved in recent years [32, 33]. However, as a result, there is a growing population of IAIs survivors, that unfortunately are now progressing into chronic critical illness with poorly defined long-term outcomes [34]. In fact, these patients may experience new symptoms, long-term disability, worsening of chronic health conditions, and increased risk for death following long hospitalization in healthcare facilities [35, 36].

Timely and, whenever possible, culture driven diagnosis, adequate source control, early and appropriate antimicrobial therapy, and expeditious physiological stabilization in critically ill patients are of paramount importance. Clinical, instrumental, and laboratory investigations should be proposed according to the clinical conditions with a step-up approach [37].

A very important issue at present is represented by the necessity to introduce the antibiotic stewardship concept and its implementation toward the reduction of antibiotic usage and misuse. This would warrant better strategies aiming to preserve antimicrobials effectiveness in next the years [38–40]. IAIs treatment encompass various combinations of strategies aiming to control the source, initiate empirical antimicrobial therapy as soon as possible and in the most severe cases hemodynamic support. A few strategies may be posed in action to contribute to restore the physiology aiming to expand the concept of source control beyond the mere surgical control of the source of infection. The therapeutical pressure variates according to the severity of the infection, to the physiologic deranging effects and to the patients baseline conditions [41, 42].

Present study aimed to obtain a picture of IAIs in Italy and their management. Up to now it represents the biggest cohort study about the topic ever realized in Italy. Even if few regions of the country have not enrolled patients into the register IRIS study covered the most part of the country. Thousands of patients with a well-balanced case mix have been enrolled. Interesting data about bacteria epidemiology and IAIs management have been obtained.

In general, one of the most impairing biases of the IAIs registries is the unbalanced enrollment of only some IAI and the prevalence of acute appendicitis over the other diseases. In present study the different infections are well-balanced, and the data are for the most of patients complete with a very small number of missing for the analyzed variables. This gave the opportunity to have a real-life picture of the cIAIs in Italy. Table 1 represents the distribution of the different cohorts of patients and all the presenting combination of IAIs are listed. The incidence of the different diseases in the different patients can be read and the known epidemiology of the several diseases is confirmed. Some diseases are mostly represented in young population, and some are more present in older people. In general, the surgical emergencies interest the people over the 5th decades of life apart from the acute appendicitis and gynecological emergencies.

The most part of infections fall under the classification of Community acquired infections (CA). This would mean that most of the bacteria would be sensible to the most of antimicrobials and for this reason easier to be treated. As already demonstrated, however, resistant bacteria are more frequent than expected in CA infections [43, 44]. The real incidence of resistances in CA bacteria is evolving and mostly unknown. Present study gives an overview of the different species isolated from cIAIs and the idea of which are the antimicrobials prescribing attitudes in Italy.

Table 2 shows how the different way to research for bacteria in surgical patients. One third of patients didn’t experience any kind of bacteria research. In many patients an intrabdominal culture was obtained with an overall positivity of 48.8%. Whenever the intra-abdominal culture was associated to a blood stream one the positivity was 50.4%. Blood stream culture alone showed a positivity of 17.5%. This data suggests associating in patients with cIAIs the intra-abdominal culture to the bloodstream to have the best possibilities of refining the antibiotic therapy. Table 3 shows the different isolated bacteria. As previously said resistant bacteria are variously mixed with the most of them isolated from the intra-abdominal cultures. To increase the antibiotic stewardship appropriateness bacteria isolation from intra-abdominal cavity is of paramount importance, better if with the association between peritoneal and blood samples.

Fungi as well are underestimated. They are unfrequently isolated as showed in Table 4. Whenever isolated, they showed a low percentage of resistances. As for bacteria, if the peritoneal and blood cultures are associated the research is more effective in isolating fungi.

Antimicrobics and antimycotics prescription is shown in Tables 5 and 6. The great variety of prescribed antimicrobials demonstrates ad antibiotic stewardship programs are largely needed in Italy. In fact, present data showed a great number of resistant bacteria in cIAIs. Many of the treated patients come from home. The number of resistant bacteria in community acquired infections is underestimated. Dedicated studies to improve knowledges about the source of resistances deriving from community acquired infections should be implemented. Lastly, last decades of antibiotic prescriptions policies brought worldwide to the number of resistances increasing year by year. Antibiotic stewardship programs implementation needs to be mandatory worldwide.

On one hand empiric antibiotic therapy was prescribed in 78.4% of patients with a median duration ranging from 5.9 to 12.9 days. Amoxicillin/clavulanate was the most common antibiotic used (in 30.1% appendicitis, 30% bowel occlusion, 30.5% of cholecystitis, 51% complicated abdominal wall hernia, 55% small bowel perforation) followed by piperacillin/tazobactam. (13.3% colonic perforation and diverticulitis, 22.6% cholecystitis, 24.2% intestinal ischemia, 28.6% pancreatitis). In general data showed as empiric antibiotic therapy is prescribed according to the severity of the disease and to the potential pathogens involved. Lastly, quinolones are generally not prescribed, and combination of molecules are reserved to those diseases that generally may present a multi bacteria aetiology.

On the other hand, however, many patients experienced empiric antimycotics prescription even in clinical conditions that generally don’t require them. Tables 6 and 7, in fact, suggest that, in general, a more appropriate prescription of antimycotics is urgently needed. Empiric antifungal therapy was administered in 2.6% of patients with no sign of sepsis, 3.1% of patients with clinical sign of sepsis and 4.1% of patients with septic shock (Table 6). Azoles was administered in 49.2% of patients that received empiric antifungal therapy (Table 7). In fact, several patients experience the use of azoles as front-line therapy, and this may be matter of debate. Antifungal as well as antibiotic prescribing attitude should be implemented. All clinicians must be aware about the necessity to know how to properly prescribe antibacterial therapies even in absence of infectious disease specialist support, as often happens in emergency settings.

Lastly ICU admission and mortality in all cIAIs is generally in the range described from the literature and for some diseases even lower demonstrating that cIAIs are well managed and treated.

## Conclusion

Complicated intra-abdominal infections in Italy are a diffuse disease. The population affected is in general over the 5th decades of life and resistant bacteria are frequently involved. Community acquired infections represent the majority of the intra-abdominal infection cases, in particular appendicitis represented the main diagnosis among CA infection. Not surprisingly, the major pathogen involved in intra-abdominal infections was found to be *E.coli*. empiric antimicrobial therapy was administered in 78.4% of patients. Looking at the data, antibiotic stewardship programs and correct antimicrobial and antimycotic prescription campaigns are necessary to ulteriorly improve the adequacy of drug usage and reduce the resistances burden. This will help in improving the care and the cure of the next generations.

## Data Availability

No datasets were generated or analysed during the current study.

## Abbreviations

IAI: Intra-abdominal infection
uIAI: Uncomplicated intra-abdominal infection
cIAI: Complicated Intra-abdominal infection
AGORA: Antimicrobials: A global alliance for optimizing their rational use in intra-abdominal infections
WSES: World society of emergency surgery
IRIS: Italian register of complicated intra-abdominal infections
MDRO: Multidrug-resistant organisms
WBC: White blood count
qSOFA: Quick sequential organ failure assessment
CA-cIAIs: Complicated IAIs will be classified as community- acquired
HA-cIAIs: Healthcare-acquired
SOFA: Sequential organ failure assessment
ICU: Intensive care unit
LOS: Length of hospital stay
CLSI: Clinical laboratory standards institute
EUCAST: European committee on antimicrobial susceptibility testing
ESBLp: *E.coli* producing an extended-spectrum b-lactamase
MRSA: Methicillin-resistant Staphylococcus aureus
